# Death from Chloroform

**Published:** 1869-07-01

**Authors:** 


### *Death from Chloroform.*

Henry Gibbons, Jr., M.D., *Pacific Med. Journal*, reporting instances of fifty-seven deaths from chloroform, concludes as follows:

Since collecting and arranging the above cases, some others have come to my knowledge, which must, however, be deferred to another occasion. Here are 57 deaths, of which 32 occurred in the United States—5 of them in 1867, and 10 in 1868—19 in England, 2 in Paris, and one each in Dublin, Vienna, Prussia and Canada. They occurred in the following years: one each in 1850, 1856, 1858, 1859, 1860 and 1865; 3 each in 1862, 1864 and 1866; 13 in 1867; 24 in 1868; and in the remaining 4 the year is not stated. Omitting the two duplicated cases in Dr. Reeves' paper there are now 188 deaths from chloroform on record, of which 104 occurred in England and Scotland, 40 in the United States, 18 in France and the rest in various other countries. The mortality in each year is as follows: 1848, 8; 1849, 6; 1850, 8; 1851, 4; 1852, 7; 1853, 5; 1854, 10; 1855, 4; 1856, 4; 1857, 2; 1858, 7; 1859, 7; 1860, 7; 1861, 6; 1862, 15; 1863, 8; 1864, 11; 1865, 5; 1866, 6; 1867, 13; 1868, 24; and in 21 the year is not stated, but at least 15 of them are known to have taken place prior to

1862. The extraordinary mortality reported for 1868, which with one exception is confined to but two countries, the United States (10) and England (13), is worthy of the most thoughtful consideration. Great interest in this subject has, of late, been manifested. The medical journals seem to have been specially active in seeking out and publishing all cases of death from the agent under consideration, and to this fact is no doubt due, at least in great part, the large number above noted. The number of deaths in 1865 and 1866 is ridiculously small, if it is proper to apply such an adjective to so serious a matter.

Highly interesting and instructive facts may be learned by careful examination and consideration of the cases presented in this paper. They have reference to the time at which death took place; the mode of dying; the means used in the endeavor to prevent death; the amount of chloroform inhaled; the previous use of the anæsthetic, etc. They mainly corroborate the results of previous investigations, and will, therefore, be considered very briefly, as the space already occupied is far greater than was anticipated.

Death in a majority of the cases was sudden. In 28 it occurred almost immediately or within a very few minutes of the exhibition of the anæsthetic, and before or immediately after the commencement of the operation; in 4 it ensued just as the operation was completed; in 17 there is no definite statement upon this point, though in the majority of them the inference is that death was sudden; and in 8 the fatal result was more or less remote from the termination of the operation, or was not due entirely to chloroform.

Of these latter, one (XII) was that of a man who died seventeen hours after he had undergone an extensive operation—excision of the scapula—and death was probably by syncope; one, (XIV) a man who lying on his back, vomited, and the fluid not being thrown out of his mouth, some of it was supposed to have been drawn into the trachea, as violent struggling ensued; he became black in the face and died at once, evedently from asphyxia; in two cases (XXV, XXVI) the fatal result was in part, at least, attributable to the inhalation of impure chloroform, as the latter was found to contain hydrochloric acid, and the symptoms were those which might be caused by the action of irritant gases or vapors upon the air passages. Death took place in twelve hours. In case XXXIX there was *stated* to be pulmonary phthisis; the patient "rallied for a time" and then expired; in case XL the patient after the operation spoke a few words, "gasped three times and was dead;" and in case XLI there was great loss of blood. Four of the deaths were in persons who took the chloroform while alone, and on their own responsibility, the length of time after the commencement of inhalation being, of course, unknown.

As regards the mode of dying, it was probably very generally by syncope, though of the 17 instances in which this fact can be ascertained, 9 were by syncope and 8 by asphyxia. The means to be used to prevent death when alarming symptoms have arisen, of course depend very much upon the mode of death. The same plan, however, seems to have been adopted in nearly all the cases, the records of which contain any mention of the subject. It consisted in drawing out the tongue; dashing cold water on the face; applying ammonia to the nostrils, though what benefit could arise from this, after cessation of breathing, is certainly not apparent; and artificial respiration, by Marshal Hall's method, by forcing air into the lungs either through the mouth or an opening in the trachea, or by other means. In a number of instances galvanism was resorted to, and in one or two the patient was bled. Over a year ago a physician in New York claimed to have saved the life of a patient who had apparently died while under the influence of chloroform, by placing his body at an angle of forty-five degrees. The description of this process was very obscure, but in all probabability it was the same as that recently proposed by Dr. E. L. Holmes, in an article in the *Chicago Medical Examiner*, *Sept.*, *1868*, entitled "Position in the Treatment of Chloroform Poisoning." In this article he advises when syncope has occurred, "to place the patient with the head downward on an inclined plane of at least 40°." The method of course would not be suitable in case of asphyxia, where there is evident congestion of the nervous centers, but only in those cases in which the brain is temporarily anemic, the gravitation of the blood overcoming this condition.

I recently administered chloroform (against my wish, but ether was absolutely refused) to a lady for a minor operation. She had been in the habit of using it for years to allay neuralgia, and had taken it in large quantities. Pouring a few drops upon a handkerchief, I placed it near the nostrils and was obliged to repeat the process so frequently, before there was loss of sensation, that over two ounces of chloroform were used. Several times I almost resolved to discontinue the chloroform, but finally anæsthesia occurred and the operation was performed. On its completion, and after the inhalation had been discontinued, the patient became deathly pale and ceased to breathe. I immediately drew the head and shoulders from the pillow on the sofa upon which she was lying, and lowered them below the level of the body, when breathing was almost directly resumed, and the head was replaced on the pillow. But breathing in a few moments ceased again, and the same process being repeated it again returned, the head thereafter being kept low until the tendency to syncope had passed. This is a fair illustration of the advantage of position in the syncope of chloroform poisoning as suggested by Dr. Holmes.

In only seventeen cases is the amount of chloroform used designated. This was a drachm in three cases, from one to two drachms in eight cases, two and a half and four and a half drachms in two cases, eight drachms in three cases and ten drachms in one case. In another case it is simply stated that a large quantity was given. Eight of the patients, it is stated, had previously inhaled the anæsthetic, and in all but one of them it had acted kindly.

In conclusion it may be added, that in view of the facts noted in this communication, we cannot felicitate ourselves as Prof. Billroth recently did when he said in a clinical lecture, "Fortunately we may regard death from chloroform as a very rare occurrence, and one becoming more rare every year."

